# Expression of Concern: The efficacy of a novel insecticidal protein, *Allium sativum* leaf lectin (ASAL), against homopteran insects monitored in transgenic tobacco

**DOI:** 10.1111/pbi.14327

**Published:** 2024-03-20

Dutta, I., Saha, P., Majumder, P., Sarkar, A., Chakraborti, D., Banerjee, S. and Das, S. (2005), The efficacy of a novel insecticidal protein, *Allium sativum* leaf lectin (ASAL), against homopteran insects monitored in transgenic tobacco. Plant Biotechnology Journal, 3: 601–611. https://doi.org/10.1111/j.1467-7652.2005.00151.x

This Expression of Concern is for the above article, published online on 16 August 2005 in Wiley Online Library (wileyonlinelibrary.com), and has been published by agreement between the journal's Editor‐in‐Chief, Johnathan Napier, the Society for Experimental Biology, the Association of Applied Biologists and John Wiley & Sons Ltd.

The Expression of Concern has been agreed due to concerns raised by a third party regarding the alleged duplication of lanes 1 and 2 in Figure 8 and a subsequent integrity investigation, that found further irregularities in Figure 9 lane 3. The authors were unable to provide a satisfactory explanation and due to the time elapsed since publication, the original raw data were not available. Consequently, the journal team could not verify the authenticity of these Figures and could not exclude that these concerns affect the overall conclusions of the article. Therefore, the journal has decided to issue an Expression of Concern to inform and alert the readers.

